# Electrophysiological Investigation of the Subcellular Fine Tuning of Sympathetic Neurons by Hydrogen Sulfide

**DOI:** 10.3389/fphar.2017.00522

**Published:** 2017-08-04

**Authors:** Manuel Dominguez-Rodriguez, Helmut Drobny, Stefan Boehm, Isabella Salzer

**Affiliations:** Department of Neurophysiology and Neuropharmacology, Center for Physiology and Pharmacology, Medical University of Vienna Vienna, Austria

**Keywords:** H_2_S, sympathetic neurons, excitability, K_*ATP*_ channels, transmitter release, K_υ_7 channels

## Abstract

H_2_S is well-known as hypotensive agent, whether it is synthetized endogenously or administered systemically. Moreover, the H_2_S donor NaHS has been shown to inhibit vasopressor responses triggered by stimulation of preganglionic sympathetic fibers. In contradiction with this latter result, NaHS has been reported to facilitate transmission within sympathetic ganglia. To resolve this inconsistency, H_2_S and NaHS were applied to primary cultures of dissociated sympathetic ganglia to reveal how this gasotransmitter might act at different subcellular compartments of such neurons. At the somatodendritic region of ganglionic neurons, NaHS raised the frequency, but not the amplitudes, of cholinergic miniature postsynaptic currents via a presynaptic site of action. In addition, the H_2_S donor as well as H_2_S itself caused membrane hyperpolarization and decreased action potential firing in response to current injection. Submillimolar NaHS concentrations did not affect currents through K_υ_7 channels, but did evoke currents through K_*ATP*_ channels. Similarly to NaHS, the K_*ATP*_ channel activator diazoxide led to hyperpolarization and decreased membrane excitability; the effects of both, NaHS and diazoxide, were prevented by the K_*ATP*_ channel blocker tolbutamide. At postganglionic sympathetic nerve terminals, H_2_S and NaHS enhanced noradrenaline release due to a direct action at the level of vesicle exocytosis. Taken together, H_2_S may facilitate transmitter release within sympathetic ganglia and at sympatho-effector junctions, but causes hyperpolarization and reduced membrane excitability in ganglionic neurons. As this latter action was due to K_*ATP*_ channel gating, this channel family is hereby established as another previously unrecognized determinant in the function of sympathetic ganglia.

## 1. Introduction

Cardiovascular disease is a leading cause of death worldwide with elevated blood pressure, raised blood lipids and glucose, as well as thrombosis and inflammation as major underlying pathophysiological risk factors (Tzoulaki et al., 2016). The proper function of the cardiovascular system relies on a variety of regulatory elements amongst which gasotransmitters play a prominent role (Li et al., 2009; Mustafa et al., 2009). While the importance of nitric oxide (NO) and carbon monoxide (CO) has been widely recognized, the contribution of a third gas, hydrogen sulfide (H_2_S) came into the focus of interest more recently (Wang, 2012). In general, all three gasotransmitters attenuate tissue damage, for instance in the myocardial ischemia reperfusion injury model, and provide protective effects in the cardiovascular system, in particular vasodilation (Li et al., 2009). As a consequence, NO donors have been used in cardiovascular pharmacotherapy for more than a century (Herman and Moncada, 2005), and drugs that elevate CO and H_2_S levels are being developed (Li et al., 2009). In particular, recent research has focused on the design of novel H_2_S donors several of which were proven to provide cardioprotective effects, even though the underlying mechanisms remained elusive (Zhao et al., 2015).

H_2_S is produced in a variety of tissues by three different enzymes, cystathionine β - synthase, cystathionine - γ - lyase, and 3 - mercaptopyruvate sulphurtransferase, as well as non-enzymatic mechanisms (Wang, 2012; Kimura, 2015). In the vasculature, cystathionine- γ -lyase is the dominating enzyme, and knock out of this protein leads to a loss of H_2_S and to hypertension (Yang et al., 2008). In the central nervous system (CNS), in contrast, H_2_S is mainly synthetized by cystathionine β - synthase and modulates glutamatergic neurotransmission and synaptic plasticity (Abe and Kimura, 1996). In the periphery as well as in the CNS, application of H_2_S donors such as NaHS causes a decrease in blood pressure, and in the brain as well as in the vasculature one major underlying mechanism is an activation of ATP sensitive K^+^ (K_*ATP*_) channels (Meng et al., 2014; van Goor et al., 2016).

A major determinant in the regulation of blood pressure is the sympathetic nervous system with both, central and peripheral components being involved (Guyenet, 2006). Effects of H_2_S or H_2_S donors on blood pressure when injected into central nuclei involved in the control of the sympathetic tone have been investigated in detail (Meng et al., 2014; van Goor et al., 2016). Moreover, systemic administration of H_2_S or H_2_S donors does not only reduce blood pressure (Meng et al., 2014; van Goor et al., 2016), but also inhibits the vasopressor response to preganglionic stimulation of the sympathetic nervous system while leaving the vasopressor response to noradrenaline and methoxamine, an α - adrenergic agonist, unaltered (Centurión et al., 2016). This suggests that H_2_S may act at the level of postganglionic sympathetic neurons, but direct effects of this gasotransmitter on such neurons have not been investigated. Nevertheless, evidence has been provided that H_2_S is synthetized in sympathetic ganglia, and two relevant enzymes, cystathionine- γ -lyase as well as cystathionine β - synthase have been detected therein (Sha et al., 2013). In addition, blockers of these enzymes were reported to inhibit ganglionic transmission, while NaHS was found to increase the same (Sha et al., 2013). Such an augmentation of neurotransmission within sympathetic ganglia by H_2_S is in sharp contrast with the inhibition of vasopressor responses triggered by preganglionic stimulation (Centurión et al., 2016), and this discrepancy led us to investigate in detail direct actions of H_2_S and H_2_S donors onto neurons within sympathetic ganglia. In primary cultures of dissociated sympathetic ganglia one gains direct experimental access to different subcellular compartments of such neurons within one culture dish; thereby, one cannot only investigate effects occurring at the somatodendritic region that is *in vivo* located within the ganglia, but also actions on noradrenaline release which otherwise takes place at sympathetic varicosities situated within the innervated tissues such as arteries (Boehm and Huck, 1997). The results obtained in such cultures reveal that H_2_S elicits opposing responses at different segments of sympathetic neurons.

## 2. Materials and methods

### 2.1. Primary cultures of rat superior cervical ganglion neurons

Primary cultures of dissociated superior cervical ganglia (SCG) were prepared as described before (Lechner et al., 2003). Sprague-Dawley rat pups were decapitated 5–10 d after birth, the ganglia were collected immediately thereafter and cut into two to three pieces. First, they were incubated in collagenase (1.5 mg ml^−1^; Sigma-Aldrich, Vienna, Austria) and dispase (3.0 mg ml^−1^; Boehringer-Mannheim, Vienna, Austria) for 30 min at 37°C, followed by trypsinization (0.25% trypsin, Worthington, Lakewood, NJ) for 15 min at 37°C. Subsequently, they were dissociated by trituration and resuspended in Dulbecco's Modified Eagle Medium containing 2.2 g l^−1^ glucose, 10 mg ml^−1^ insulin, 25,000 IU l^−1^ penicillin, 25 mg l^−1^ streptomycin (all Sigma-Aldrich), 50 μg l^−1^ nerve growth factor (Biomedica, Vienna, Austria), and 5% heat-inactivated fetal bovine serum (Biochrom, MB Stricker, Tutzing, Germany). Thereafter, cells were seeded onto 35 mm dishes (Nunclon, Bartelt, Graz, Austria) for electrophysiological experiments or 5 mm plastic discs for radiotracer release experiments, both coated with rat tail collagen (Trevigen, Szabo-Scandic, Vienna, Austria). The cultures were kept at 37°C in a humidified 5% CO_2_ atmosphere for 4–8 days for all types of experiments with one exception: for recordings of cholinergic miniature excitatory postsynaptic current (mEPSCs), cultures were kept for 3 weeks (O'Lague et al., 1974). On days one and four after preparation, the medium was exchanged entirely. The medium of long-term cultures was replaced once per week, and in week 2 after preparation 1 μM cytosine arabinoside (Ara-C, Sigma-Aldrich) was added to the culture medium to prevent proliferation of non-neuronal cells.

### 2.2. Electrophysiology

Electrophysiological recordings were performed at room temperature (20–24°C) using an Axopatch 200B amplifier, a Digitata 1320 interface and the Clampfit 10.2 software (Molecular Devices, Sunnyvale, CA). All current- and voltage-clamp recordings were performed in the perforated-patch mode. mEPSCs were low-pass filtered at 5 kHz and digitized at 10 kHz, currents through nicotinic acetylcholine receptors were low-pass filtered at 10 kHz and sampled at 20 kHz, all other recordings were low-pass filtered at 2 kHz and stored at 10 kHz. The data were analyzed offline using the Clampex 10.4 software (Molecular Devices). Patch-pipettes were pulled from borosilicate glass capillaries (GB150-8P, Science Products, Hofheim, Germany) with a Sutter P-97 puller (Sutter Instruments, Novato, CA) using a trough filament (FT330B, Science Products). Pipette resistances were between 1.5 to 3.5 MΩ. Pipettes were front-filled with internal solution and then back-filled with the same solution containing 500 μg ml^−1^ amphotericin B. Recordings were started as soon as the series resistance had stabilized and dropped below 20 MΩ, typically after 20 to 30 min.

The internal solution for all electrophysiological recordings contained (in mM): K-gluconate (133), NaCl (5.9), CaCl_2_ (1), MgCl_2_ (0.7), HEPES (10), EGTA (10), and KOH (29.4) adjusted to pH 7.4. The external solution for all electrophysiological recordings contained (in mM): NaCl (140), glucose (20), HEPES (10), CaCl_2_ (2.5), MgCl_2_ (2.0), KOH (3.0) and NaOH (2.0) adjusted to pH 7.4. The external solution for evoking mEPSCs contained (in mM): NaCl (120), KCl (20), glucose (20), HEPES (10), CaCl_2_ (2.5), MgCl_2_ (2.0), KOH (2.5), NaOH (2.5) adjusted to pH 7.4. Additionally, in all voltage-clamp recordings tetrodotoxin (0.5 μM) was included in the bath solution. These solutions resulted in a liquid junction potential of 15.6 mV which was corrected for during measurements. When currents through nAChRs were recorded, drugs were applied using a piezo-switched fast-step perfusion (SF-77B, Warner Instruments, Hamden, CT) connected to two gravity-driven eight-channel valve control systems (VC-8 system, Warner Instruments). In all other recordings, drugs were applied using a DAD-12 drug application device (ALA Scientific Instruments Inc., Westbury, NY), which permits complete exchange of solutions surrounding the recorded cells within 50 to 150 ms.

In order to evaluate changes in membrane excitability, cells were injected with five increasing 1 s current steps from 50 pA to 250 pA, in 50 pA increments, once every 12 s. The cells were exposed to solvent for 180 to 300 s before recording control values. Thereafter, modulators such as different concentrations of NaHS (0.1, 0.3, or 1 mM), H_2_S (0.1, 0.3, or 1 mM), or diazoxide (300 μM) were applied for 300 s before evaluation of changes induced by either of these agents. When appropriate, the K_*ATP*_ channel blocker tolbutamide (100 μM) was present for 120 s before co-application together with NaHS or diazoxide.

For recording currents through K_υ_7 channels, cells were clamped to −30 mV; once every 15 s the cells were hyperpolarized to −55 mV for 1 s periods; differences in amplitudes 20 ms after the beginning and 20 ms before the end of such deactivation steps were taken as a measure of such currents. Varying concentrations of NaHS were perfused for 420 s, subsequent to a control period of 90 s and followed by a 500 s wash out period where solvent was present.

Currents induced by diazoxide (0.3 mM) or NaHS (0.3 mM) were recorded at voltages corresponding to the resting membrane potentials of the neurons under investigation, which had been determined in current-clamp mode beforehand. Diazoxide or NaHS were present for 300 s, preceded and followed by 300 s periods of solvent application (0.5% DMSO–0.5% NaOH). The K_*ATP*_ antagonist tolbutamide was applied as described above for current clamp measurements. Effects of diazoxide and NaHS on steady-state currents in the absence and presence of tolbutamide were recorded in individual cells as the action of at least H_2_S may be irreversible.

For mEPSC recordings, neurons were clamped at −70 mV and a solution containing 20 mM K^+^ was applied to increase their frequency which is otherwise exceedingly low (Liu et al., 2000). Sixty seconds periods of high K^+^ were alternated with 60 s periods of regular external solution to prevent exhaustion of vesicle exocytosis. Varying concentrations of NaHS (0.1 mM, 0.3 mM, or 1 mM) were applied for 300 s. At the end of the recordings, hexamethonium (100 μM) was applied in high K^+^ solution to verify the measurement of cholinergic mEPSCs. Currents through nicotinic acetylcholine receptors (nAChRs) were evoked by 1 s applications of different concentrations of 1,1-dimethyl-4-phenylpiperazinium iodide (DMPP; 0.01 or 0.1 mM) or acetylcholine (ACh; 0.01 or 0.1 mM) to neurons clamped at −70 mV; NaHS (1 mM) was present for periods of 300 s again.

### 2.3. Determination of [^3^H]-noradrenaline release

[^3^H]-noradrenaline release was performed as described previously (Lechner et al., 2003). In brief, plastic discs with dissociated SCG neurons were incubated at 37°C for 1 h in 0.05 μM [^3^H]-noradrenaline (specific activity 42.6 Ci mmol^−1^) in culture medium containing ascorbic acid (1 mM). Subsequently, the discs were transferred into small chambers and superfused with a solution containing (in mM): NaCl (120), KCl (3.0), CaCl_2_ (2.0), MgCl_2_ (2.0), glucose (20), HEPES (10), fumaric acid (0.5), Na-pyruvate (5.0), and ascorbic acid (0.57), adjusted to pH 7.4 with NaOH. Superfusion was carried out at 25°C at a rate of about 1.0 ml min^−1^. After a wash out period of 60 min to remove excess radioactivity, 4-min superfusate fractions were collected. [^3^H]-noradrenaline release was triggered twice per experiment at minutes 72 and 92 of perfusion by one of the following three stimulation paradigms: (i) 60 monophasic rectangular pulses (0.5 ms, 60 mA, 50 V cm^−1^) delivered at 1 Hz (electrical field stimulation: EFS); (ii) application of a buffer containing 40 mM KCl (NaCl was reduced accordingly to avoid osmotic differences) for 60 s; (iii) 60 s presence of a buffer containing additional 0.5 M sucrose as hypertonic shock. The buffer used for the hypertonic shock was also deficient of any calcium. From minute 84 after the start of perfusion onward, the buffer solution was switched to one containing either solvent or variable concentrations of NaHS (0.01, 0.1, or 1 mM) or H_2_S (0.01, 0.1, or 1 mM). The residual radioactivity after completion of the experiments was extracted by immersion of the discs in 2% (v/v) perchloric acid. The radioactivity in the extracts and the collected 4-min fractions was determined by liquid scintillation counting (Packard Tri-Carb 2800 TR, PerkinElmer, Brunn/ Geb., Austria) with a counting efficiency of 63%. Radioactivity released in response to electrical field stimulation from rat sympathetic neurons after labeling with [^3^H]-noradrenaline under conditions similar to those in the present study was shown to consist predominantly of the actual neurotransmitter and to contain only less than 15% metabolites (Schwartz and Malik, 1993). Thus, tritium outflow was considered to be predominantly that of noradrenaline and not that of metabolites.

The unstimulated (spontaneous) rate of [^3^H] outflow was evaluated by expressing the radioactivity of a collected 4-min fraction as percentage of the total radioactivity present in the cultures at the beginning of the corresponding collecting period (% of total radioactivity). The stimulated [^3^H] overflow was calculated as the difference between total tritium outflow during and after the stimulation and the estimated basal outflow, which was assumed to decline linearly throughout the experiment. Hence, the basal outflow during stimulation periods was calculated as the arithmetic mean of the basal [^3^H] outflow from the fractions immediately before and after the stimulations. As evoked [^3^H] release varies considerably between different SCG preparations (Lechner et al., 2005), tritium overflow in the absence and presence of NaHS, respectively, was always compared within the same preparation. The values obtained in presence of NaHS were expressed as percentage of the corresponding control values within the same preparation.

### 2.4. Statistics

Unless specified otherwise, all values are arithmetic means ± standard error of the mean. Amplitudes of mEPSCs are weighted arithmetic means ± weighted standard deviation. *n* values reflect single cells in electrophysiological experiments and numbers of cultures in radiotracer release experiments. Statistical significance of differences between two groups was determined by the Mann-Whitney test. Statistical significance of differences between multiple groups was assessed by Kruskal-Wallis tests followed by Dunn's multiple comparison tests. *P*-values < 0.05 were considered as indicating statistical significance.

### 2.5. Materials

(-)-[Ring-2,5,6-^3^H]noradrenaline was obtained from PerkinElmer (Vienna, Austria); 1,1-dimethyl-4-phenylpiperazinium iodide (DMPP), acetylcholine, amphotericin B, diazoxide, H_2_SO_4_, hexamethonium, Na_2_S, NaHS, tolbutamide and bulk chemicals were purchased from Sigma-Aldrich. Tetrodotoxin (TTX) was obtained from Latoxan (Valence, France). Stock solutions of acetylcholine, DMPP, hexamethonium, and NaHS were prepared in deionized water, tolbutamide was dissolved in a mixture of 50% DMSO and 50% NaOH.

Gaseous H_2_S was applied as an aqueous solution saturated with H_2_S gas. The gas was produced by mixing aqueous Na_2_S [1 M] and H_2_SO_4_ [1.4 M], which produces Na_2_SO_4_ and H_2_S gas according to the formula:

(1) $${\text{Na}}_{\text{2}}\text{S}+{\text{H}}_{\text{2}}{\text{SO}}_{\text{4}}⇄{\text{Na}}_{\text{2}}{\text{SO}}_{\text{4}}+{\text{H}}_{\text{2}}\text{S}.gas$$

The liberated H_2_S gas was subsequently bubbled into a buffer containing (in mM): HEPES (124), NaCl (120), CaCl_2_ (2.0), MgCl_2_ (2.0), KCl (3.0) adjusted to pH 7.4 with NaOH to produce a saturated (93 mM) stock solution. To ensure saturation, a six fold exess of H_2_S gas was produced and bubbled into 100 ml buffer. Evaporated gas was trapped in a washing bottle containing H_2_O_2_ in order to transform H_2_S into H_2_SO_4_. The saturated stock solution had to be used immediately afer production, as the H_2_S concentration dropped to about 20 mM after only 3 days in solution. Dilute concentrations of H_2_S were exchanged regularly and used for up to 1 h only.

### 2.6. Ethics approval statement

This study was carried out in accordance with the guidelines of “Good Scientific Practice” of the Medical University of Vienna and with all the rules of the Austrian animal protection law (http://www.ris.bka.gv.at/Dokumente/BgblAuth/BGBLA_2012_I_114/BGBLA_2012_I_114.pdf) and the Austrian animal experiment bylaws (http://www.ris.bka.gv.at/Dokumente/BgblAuth/BGBLA_2012_II_522/BGBLA_2012_II_522.pdf) which implement the European directive (http://eur-lex.europa.eu/LexUriServ/LexUriServ.do?uri=OJ:L:2010:276:0033:0079:en:PDF) in Austrian law.

The experiments were carried out *ex vivo*, wild type animals were sacrificed and their ganglia were thereafter used for experiments. §2 of the Austrian animal protection law (http://www.ris.bka.gv.at/Dokumente/BgblAuth/BGBLA_2012_I_114/BGBLA_2012_I_114.pdf) states that such experiments do not require an approval of the institutional ethics committee.

## 3. Results

### 3.1. H_2_S and NaHS decrease membrane excitability in superior cervical ganglion neurons

**Figure 1:**
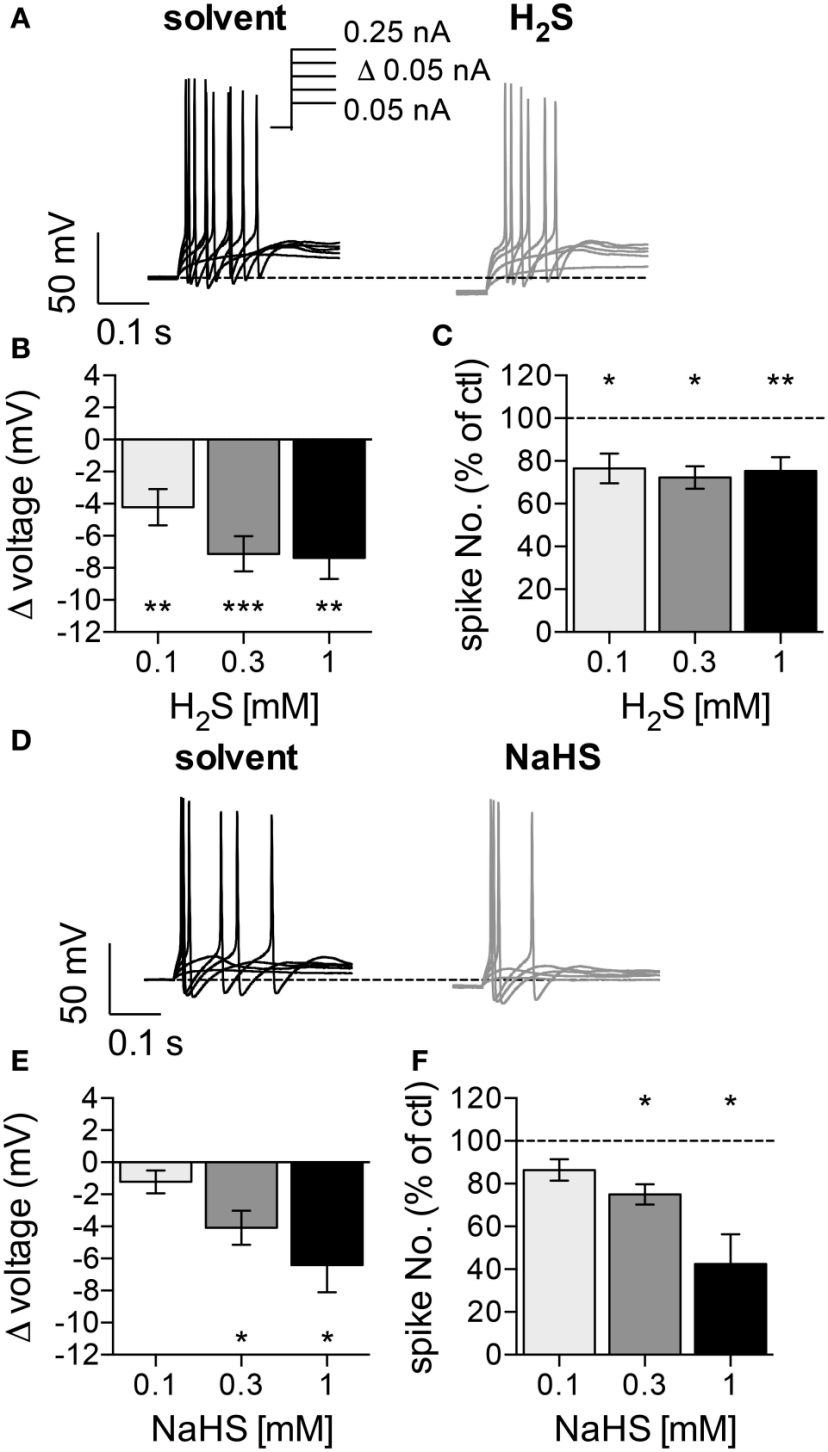
Effects of H_2_S and NaHS on membrane potential and action potential firing in SCG neurons. The membrane potential was recorded in the perforated current-clamp mode. Currents with increasing amplitudes (50 to 250 pA as indicated in a) were injected for 1 s periods, once every 12 s. The membrane potential was evaluated during the 1 s interval before current injection. Baseline values were recorded during a 5 min application of solvent, followed by a 5 min application of either H_2_S (0.1, 0.3, 1 mM) or NaHS (0.1, 0.3, 1 mM). **(A)** Representative traces were recorded in the presence of solvent (black trace) and 0.3 mM H_2_S (gray trace), respectively. **(B)** Changes in membrane voltage induced by 0.1 mM (*n* = 9), 0.3 mM (*n* = 11), and 1 mM (*n* = 8) H_2_S, respectively. **(C)** Changes in the number of action potentials triggered by current injections in the presence of 0.1 mM (*n* = 9), 0.3 mM (*n* = 11), and 1 mM (*n* = 8) H_2_S, respectively. The number of spikes in the presence of H_2_S is shown as percentage of the number of spikes in solvent. **(D)** Representative traces were obtained in the presence of solvent (black trace) and 0.3 mM NaHS (gray trace), respectively. **(E)** Changes in membrane voltage induced by different concentrations of NaHS (*n* = 8). **(F)** Changes in the number of action potentials triggered by current injections in the presence of different NaHS concentrations. The number of spikes in the presence of NaHS is shown as percentage of the number of spikes in solvent (*n* = 8). ^*^, ^**^, ^***^ Indicate significant differences vs. control at *p* < 0.05, *p* < 0.01, and *p* < 0.001, respectively (Wilcoxon matched-pairs signed rank test).

To assess whether hydrogen sulfide (H_2_S) affects the membrane potential and/or action potential firing in SCG neurons, cells were recorded in the perforated current-clamp mode to maintain intracellular signaling cascades potentially involved in the actions of the gas. Currents with increasing amplitudes (50 pA to 250 pA in 50 pA increments) were injected for 1 s periods once every 12 s (Figure 1A), and the neurons fired 25 ± 7 action potentials in response to these stimuli (*n* = 20). The resting membrane potential prior to current injection amounted to −72.4 ± 1.2 mV (*n* = 20), which corresponded well to the estimated membrane potential of −74.13 mV obtained via the Goldman-Hodgkin-Katz equation with K^+^, Na^+^, and Cl^−^ concentrations of our solutions and relative permeabilities for these ions of 1 : 0.04 : 0.1 (Brown and Scholfield, 1974). Solutions containing 0.1 mM to 1 mM H_2_S, when present for 5 min, hyperpolarized the membrane potential by 4 to 8 mV (Figure 1B) and reduced action potential firing triggered by current injection by up to 30% (Figure 1C).

To facilitate experimentation, the H_2_S donor NaHS was used instead of the gas. H_2_S is steadily liberated from this donor, and the H_2_S concentrations achieved in physiological salt solutions amount to approximately 25% of that of NaHS itself (Sitdikova et al., 2014). At 0.1 mM, NaHS failed to cause any significan alteration. At higher concentrations (0.3 mM and 1 mM), NaHS also caused hyperpolarization by up to 7 mV (Figure 1E) and decreased action potential firing by up to 60% (Figure 1F). Nevertheless, the relative changes in membrane potential and spike firing induced by identical concentrations of either H_2_S or NaHS were not significantly different from each other (*p* > 0.05; Kruskal-Wallis analysis followed by Dunn's multiple comparisons tests). Thus, H_2_S and NaHS affect the membrane potential and excitability of SCG neurons in a similar manner, but the minimum concentrations of NaHS required to cause significant alterations are at least threefold higher than those of H_2_S.

### 3.2. High NaHS concentrations reduce currents through K_υ_7 channels

**Figure 2:**
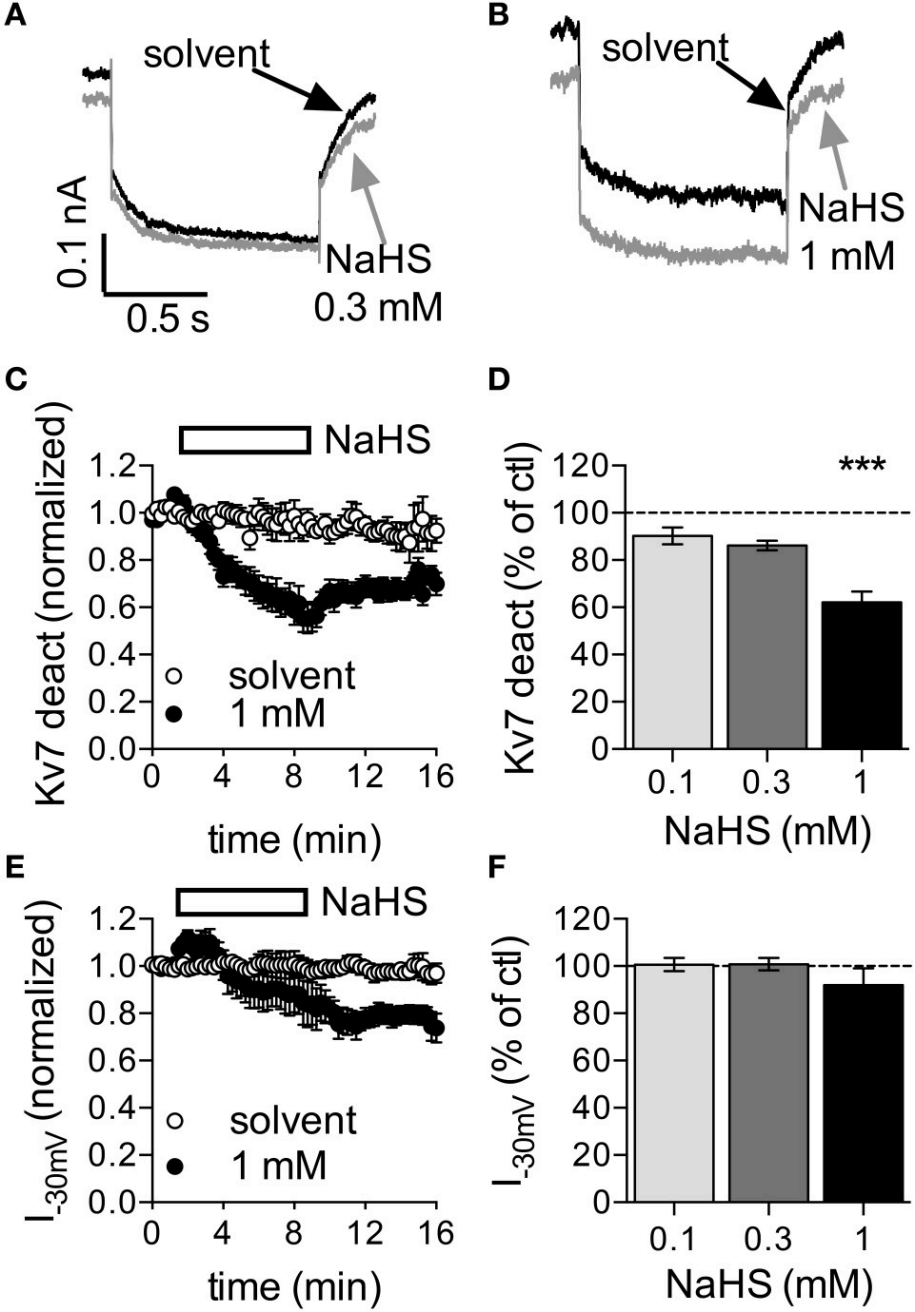
Effect of NaHS on currents through K_υ_7 channels in rat SCG neurons. Currents through K_υ_7 channels were recorded in the perforated voltage-clamp mode. The cells were clamped to −30 mV and hyperpolarized to −55 mV for 1 s periods once every 15 s. Differences between amplitudes determined at the beginning and at the end of this hyperpolarization-induced current deactivation (K_υ_7 deact) were taken as a measure of K_υ_7 currents. NaHS or solvent were applied for periods of 450 s as depicted in **(C,D)**. **(A,B)** Original current traces obtained before (black trace, **A,B**) and at the end of the application of either **(A)** 0.3 mM NaHS (gray trace) or **(B)** 1 mM NaHS (gray trace), respectively. Panel **(C)** shows the time course of current deactivation amplitudes in the presence of solvent and 1 mM NaHS, respectively. NaHS (*n* = 10) or solvent (*n* = 6) were present as indicated by the rectangle. All amplitudes were normalized to the average of the first three current amplitudes. Panel **(D)** shows deactivation current amplitudes in the presence of the indicated concentrations of NaHS as percentage of the amplitudes in the presence of solvent, both determined at the end of the 450 s application period; *n* = 10 for each concentration. Panel **(E)** shows the time course of current amplitudes at −30 mV in the presence of solvent and 1 mM NaHS, respectively. NaHS (*n* = 10) or solvent (*n* = 6) were present as indicated by the rectangle. All amplitudes were normalized to the average of the first three current amplitudes. Panel **(F)** shows current amplitudes at −30 mV in the presence of the indicated concentrations of NaHS as percentage of the amplitudes in the presence of solvent, both determined at the end of the 450 s application period; *n* = 10 for each concentration. ^***^ Indicates a significant difference vs. solvent at *p* < 0.001 (Kruskal-Wallis test, Dunn's multiple comparison *post-hoc* test).

SCG neurons express K_υ_7.2 and K_υ_7.3 subunits to generate the so-called neuronal M-currents (Wang et al., 1998), which were found to regulate action potential firing and to control the membrane potential (Jones et al., 1995). Hence, we assessed the effect of NaHS on currents through K_υ_7 channels in SCG neurons. Using the perforated voltage-clamp mode to prevent rundown of the current, the cells were clamped to −30 mV to desensitize other voltage-gated ion channels. Once every 15 s, the cells were hyperpolarized to −55 mV to elicit slow deactivation of K_υ_7 channels (Boehm, 1998). NaHS at 0.1 to 0.3 mM affected neither the currents determined at −30 mV, nor the amplitudes of deactivation currents measured upon hyperpolarization to −55 mV (Figures 2A,C–F). In the presence of 1 mM NaHS, both types of current amplitudes decreased slowly and hardly recovered from inhibition after washout of the H_2_S donor (Figures 2B,C–F). Therefore, NaHS concentrations lower than 1 mM are unlikely to change currents through K_υ_7 channels in sympathetic neurons. Higher concentrations may cause an inhibition, but this latter effect leads to membrane depolarization rather than hyperpolarization (Jones et al., 1995).

### 3.3. NaHS induces currents through K_*ATP*_ channels

**Figure 3:**
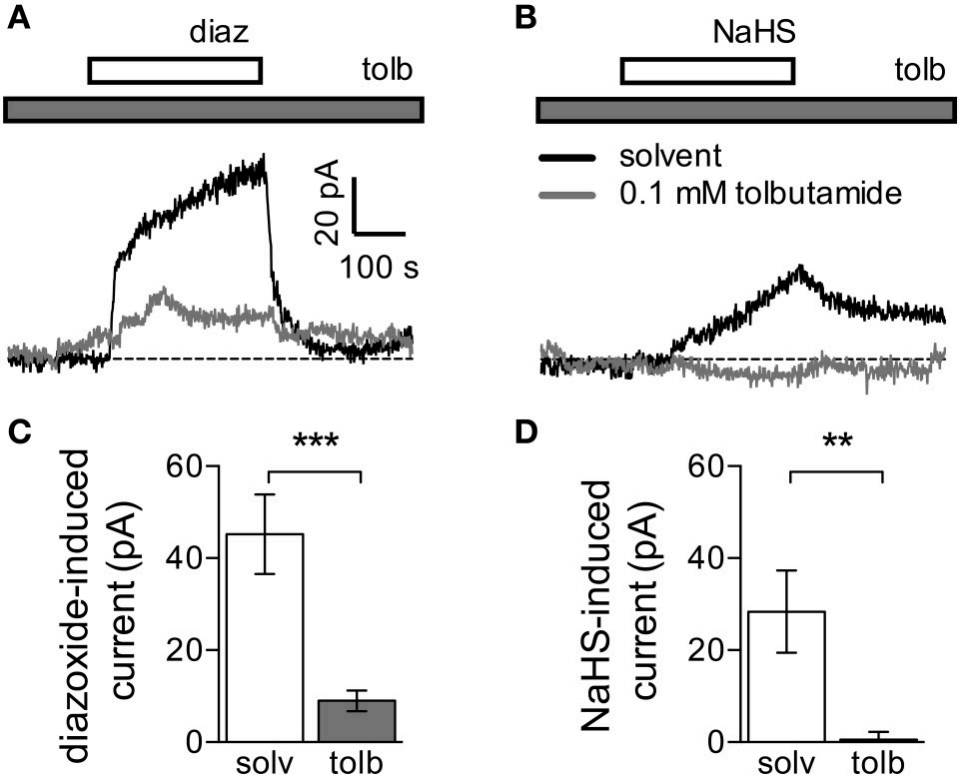
Currents induced by NaHS and the K_*ATP*_ channel activator diazoxide. Currents were recorded in the perforated voltage-clamp mode at voltages corresponding to the resting membrane potential. The latter was determined in current-clamp mode, and then the cells were voltage-clamped at the respective value. NaHS (0.3 mM) or diazoxide (0.3 mM) were applied for periods of 300 s, in the continuous presence of either solvent (0.5% DMSO + 0.5% NaOH) or tolbutamide (0.1 mM). Panels **(A,B)** show original traces of currents induced by either **(A)** diazoxide (diaz) or **(B)** NaHS in the continuous presence of either solvent (black trace) or tolbutamide (tolb, gray trace). Solvent and tolbutamide were preapplied 120 s before the start of diazoxide or NaHS, respectively. **(C,D)** Amplitudes of currents induced by **(C)** diazoxide (*n* = 8) or **(D)** NaHS (*n* = 5) in the presence of solvent (solv) and tolbutamide (tolb), respectively. ^**^ and ^***^ Indicate significant differences between amplitudes at *p* < 0.01 and *p* < 0.001, respectively (Mann-Whitney test).

As currents through K_υ_7 channels were not affected by NaHS concentrations that were sufficient to cause membrane hyperpolarization, NaHS must have acted through alternative means. To identify alternative ionic mechanisms, neurons were clamped at a voltage value corresponding to their resting membrane potential. In the presence of 0.3 mM NaHS, outward currents developed slowly and reached amplitudes of about 30 pA after 5 min. Upon removal of NaHS, current amplitudes declined but did not return to the zero current level (Figure 3A). Since K_*ATP*_ channels are well-known targets for H_2_S (Peers et al., 2012), the K_*ATP*_ channel opener diazoxide (0.3 mM) was applied instead of NaHS. Diazoxide also triggered outward currents, which reached amplitudes of about 50 pA after 5 min. After termination of the diazoxide application, outward currents rapidly dissipated (Figure 3C). To prove that the currents triggered by either NaHS or diazoxide were mediated by K_*ATP*_ channels, experiments were repeated in the continuous presence of the K_*ATP*_ channel blocker tolbutamide (0.1 mM), which largely reduced the amplitudes of currents elicited by either of the two agents (Figures 3B,D). Hence, NaHS is able to induce currents through K_*ATP*_ channels in sympathetic neurons.

### 3.4. Activation of K_*ATP*_ channels decreases membrane excitability in SCG neurons

**Figure 4:**
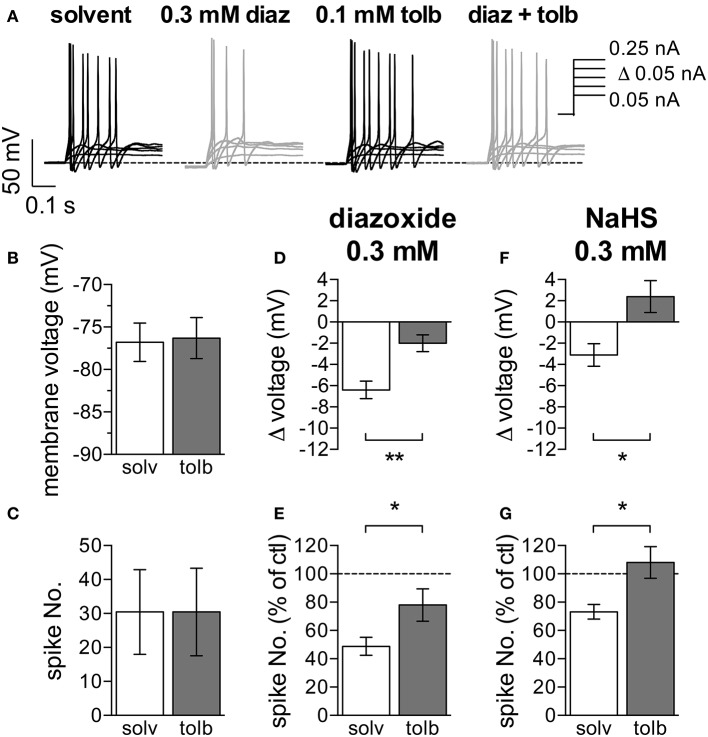
Effects of K_*ATP*_ channel modulators on membrane potential and action potential firing in SCG neurons. The membrane potential was recorded in the perforated current-clamp mode. Currents with increasing amplitudes (50 to 250 pA as indicated in **A**) were injected for 1 s periods, once every 12 s. The membrane potential was evaluated during the 1 s interval before current injection. Baseline values were recorded in presence of solvent (0.5% DMSO–0.5% NaOH), followed by a 300 s application of either diazoxide (0.3 mM, diaz) or NaHS (0.3 mM). These two agents were applied in solutions containing solvent and tolbutamide (0.1 mM, tolb), respectively, which were also present for 120 s prior to the inclusion of diazoxide or NaHS. Panel **(A)** shows representative traces recorded in the presence of solvent, diazoxide, tolbutamide, and diazoxide plus tolbutamide, respetively. Panels **(B,C)** show the effects of tolbutamide on **(B)** the membrane potential and **(C)** action potential firing (*n* = 11). **(D)** Changes in membrane voltage induced by 0.3 mM diazoxide in the presence of solvent (*n* = 10) and tolbutamide (*n* = 10), respectively. **(E)** Changes in the number of action potentials triggered by current injections due to the application of 0.3 mM diazoxide in either solvent (*n* = 10) or tolbutamide (*n* = 10). The number of spikes in the presence of diazoxide is shown as percentage of the number of spikes in in its absence. **(F)** Changes in membrane voltage induced by 0.3 mM NaHS in the presence of solvent (*n* = 6) and tolbutamide (*n* = 7), respectively. **(G)** Changes in the number of action potentials triggered by current injections due to the application of 0.3 mM NaHS in either solvent (*n* = 6) or tolbutamide (*n* = 7). The number of spikes in the presence of NaHS is shown as percentage of the number of spikes in in its absence. ^*^ and ^**^ Indicate significant differences between the values obtained in solvent and tolbutamide at *p* < 0.05 and *p* < 0.01, respectively (Mann-Whitney test).

To test if the changes in membrane excitability caused by NaHS might also involve K_*ATP*_ channels, diazoxide and tolbutamide were used in current clamp experiments as well. The K_*ATP*_ channel blocker tolbutamide (0.1 mM) on its own neither changed the resting membrane potential, nor did the drug affect action potential firing (Figures 4A–C). Conversely and in line with the results obtained with H_2_S and NaHS (Figure 1), the K_*ATP*_ channel activator diazoxide (0.3 mM) did cause hyperpolarization and did reduce action potential firing (Figures 4A,D,E). These two diazoxide-evoked effects were largely attenuated when experiments were repeated in the continuous presence of tolbutamide (0.1 mM; Figures 4A,D,E). In analogy to the results seen with diazoxide, hyperpolarization and inhibition of action potential firing caused by NaHS (0.3 mM) were both prevented in the presence of tolbutamide (0.1 mM; Figures 4F,G). Thus, the H_2_S -dependent decrease in membrane excitability relies on an activation of currents through K_*ATP*_ channels.

### 3.5. NaHS enhances cholinergic transmission in SCG neurons

**Figure 5:**
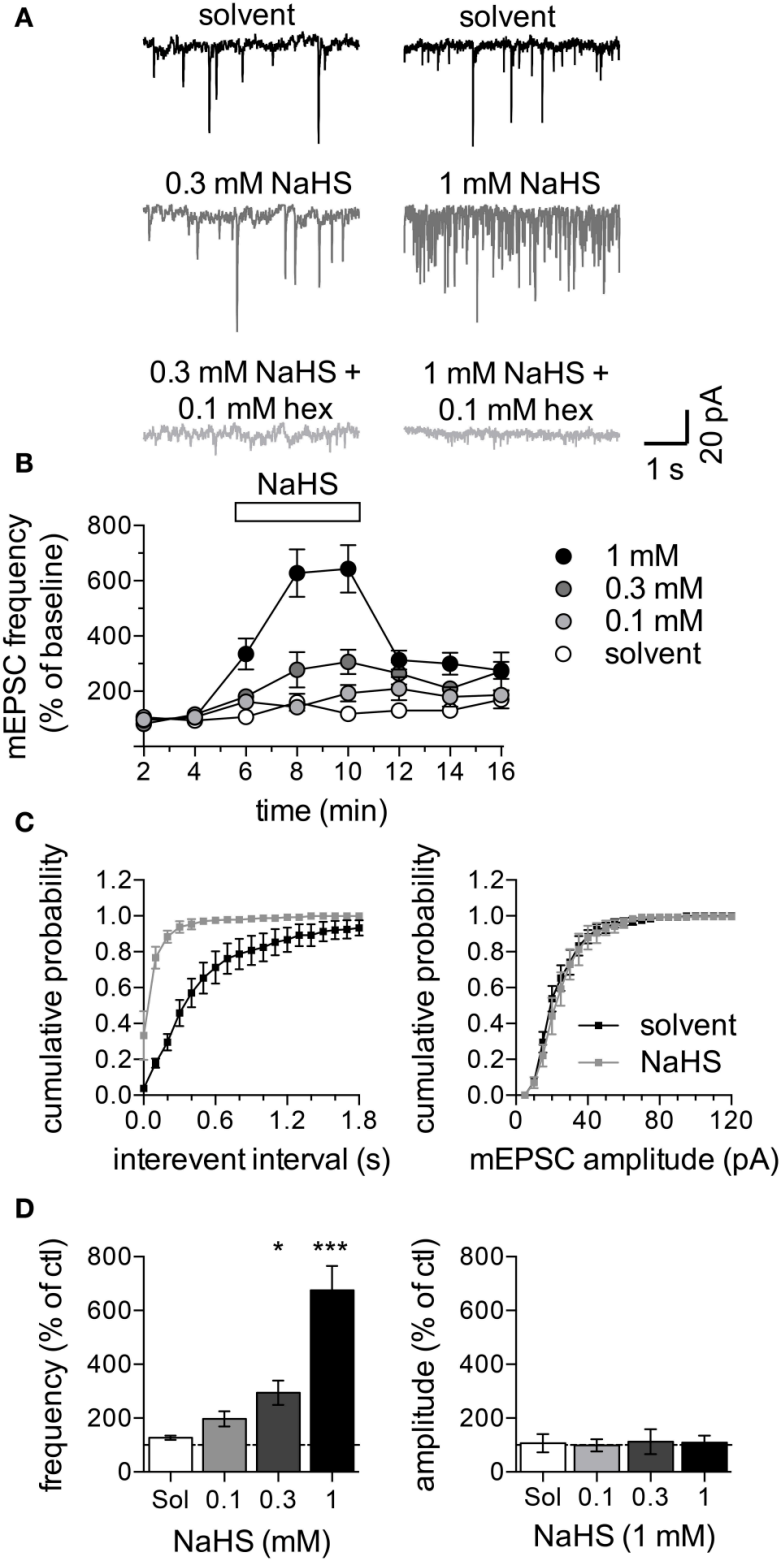
Effect of NaHS on mEPSCs in SCG neurons. mEPSCs were recorded in the perforated voltage-clamp mode in presence of 20 mM K^+^. **(A)** Representative current traces were recorded in the presence of solvent (black traces, top), 0.3 mM NaHS (dark gray, left) or 1 mM NaHS (dark gray, right) or either concentration of NaHS together with 0.1 mM hexamethonium (light gray, bottom). Panel **(B)** shows the time course of changes in mEPSC frequency in response to solvent (empty circles) or different concentrations of NaHS (0.1 to 1 mM, as indicated) which were present for 300 s as indicated by the rectangle. mEPSC frequencies are shown as percentage of the average control mEPSC frequencies recorded before the application of NaHS (*n* = 8). Panel **(C)** shows cumulative frequency distributions of mEPSC interevent intervals (left panel) and amplitudes (right panel) in presence of solvent (black squares) or NaHS (1 mM, gray squares) (*n* = 8). Panel **(D)** shows changes of mEPSC frequencies (left panel) and amplitudes (right panel) in response to solvent or different concentrations of NaHS (*n* = 8) determined at the end of the 300 s application period. ^*^ and ^***^ Indicate significance differences vs. solvent at *p* < 0.05 and *p* > 0.001, respectively (Kruskal-Wallis test, Dunn's Multiple comparison *post-hoc* test).

To reveal whether H_2_S might affect the input of sympathetic neurons, cultures of SCG neurons were maintained for up to 3 weeks to enable the formation of cholinergic synapses which can then be characterized by measuring mEPSCs (O'Lague et al., 1974). Since spontaneous mEPSCs occur infrequently in primary cultures of SCG (Liu et al., 2000), a solution containing 20 mM K^+^ was applied to neurons clamped at −70 mV; this can be expected to depolarize the presynaptic membrane to about −50 mV. This presynaptic membrane depolarization raised the mEPSC frequency to 1.5 ± 0.2 Hz (*n* = 32; Figure 5A). These K^+^-evoked postsynaptic currents were completely suppressed by hexamethonium (0.1 mM; Figure 5A), which proves that they were mediated by nAChRs. In the presence of 0.1 to 1 mM NaHS, the mEPSC frequency was reversibly increased in a concentration-dependent manner, whereas mEPSC amplitudes remained unchanged (Figures 5A–D).

**Figure 6:**
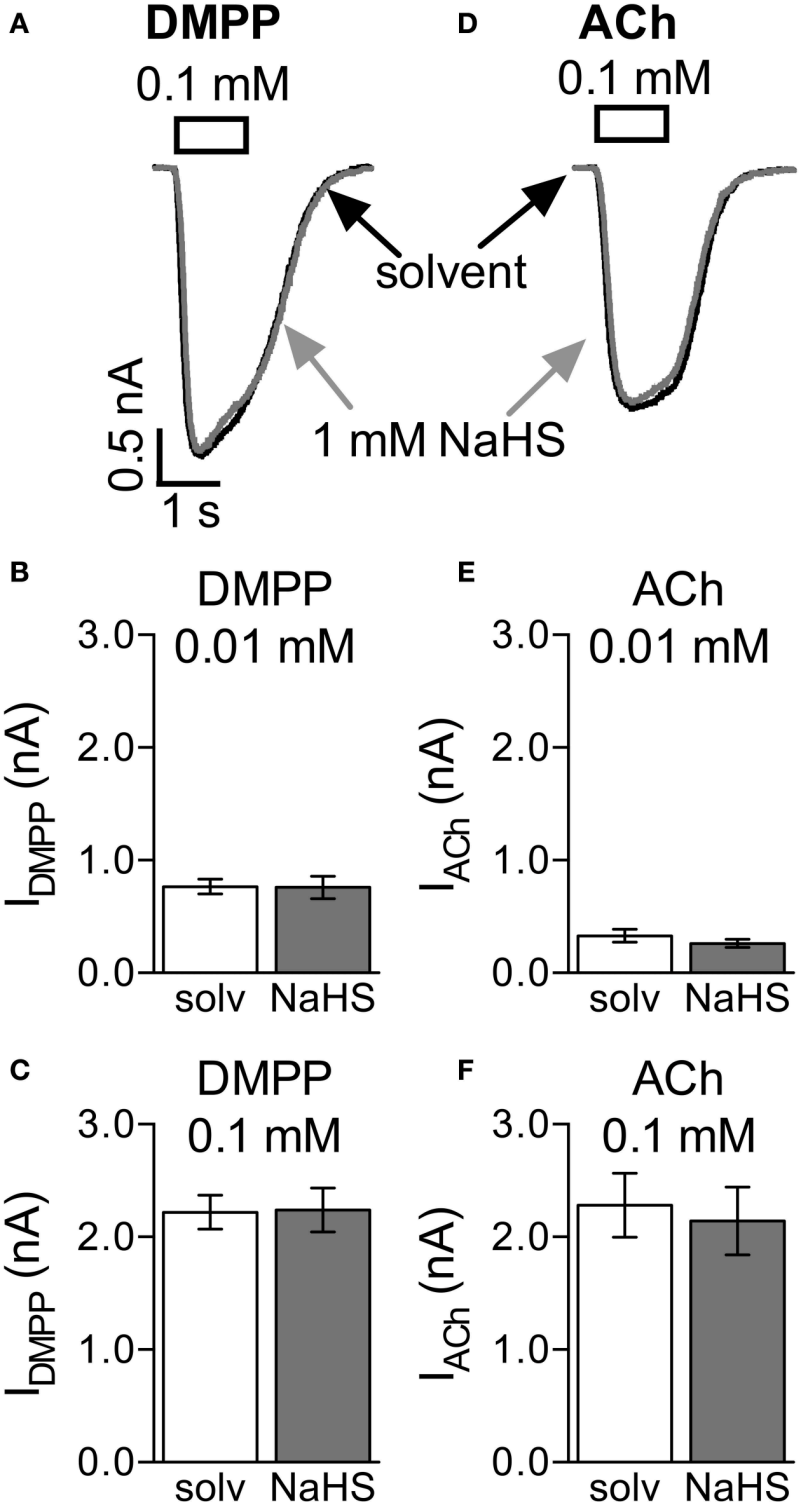
Lack of effect of NaHS on nicotinic acetylcholine receptors (nAChRs). Currents evoked by 1 s applications of DMPP (0.1 mM) or acetylcholine (ACh, 0.1 mM) were recorded in the perforated voltage-clamp mode at −70 mV. NaHS (1 mM) was present for 300 s prior to the application of nAChR agonists. Panel **(A)** shows representative traces of currents evoked by 0.1 mM DMPP in the presence of either solvent (black trace) or NaHS (1 mM, gray trace). Panels **(B,C)** show the effect of NaHS (1 mM) on maximal amplitudes of currents evoked by either **(B)** 0.01 mM (*n* = 8) or **(C)** 0.1 mM DMPP (*n* = 7). Panel **(D)** shows representative traces of currents evoked by 0.1 mM ACh in the presence of either solvent (black trace) or NaHS (1 mM, gray trace). Panels **(E,F)** show the effect of NaHS (1 mM) on maximal amplitudes of currents evoked by either **(B)** 0.01 mM (*n* = 8) or **(C)** 0.1 mM ACh (*n* = 7). No significant differences between current amplitudes were determined for solvent and NaHS, respectively (Wilcoxon matched-pairs signed rank test).

This pattern of changes in mEPSCs is compatible with a presynaptic action of NaHS. To confirm that NaHS does not act on nAChRs directly, SCG neurons were voltage-clamped to −70 mV and perfused with either 1,1 dimethyl-4-phenylpiperazinium iodide (DMPP, 0.01 mM and 0.1 mM) or acetlylcholine (ACh, 0.01 mM and 0.1 mM; Figures 6A,D). Exposure of the neurons to 1 mM NaHS for 300 s did not influence currents through nAChRs triggered by either of the two agonists (Figures 6A–F).

### 3.6. NaHS increases noradrenaline release from SCG neurons

**Figure 7:**
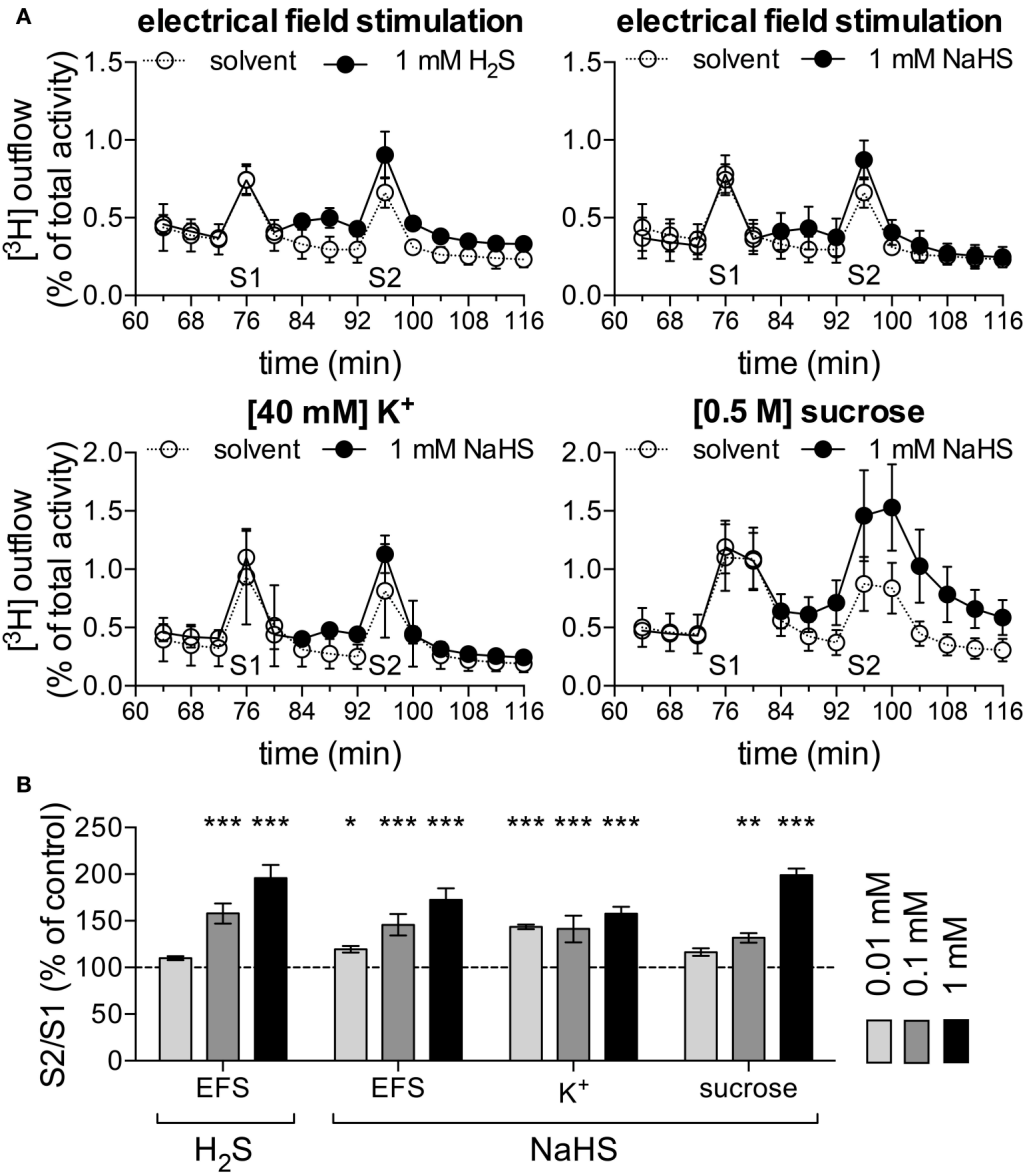
H_2_S and NaHS increase noradrenaline release. Primary cultures of rat SCG neurons were labeled with [^3^H]-noradrenaline (NA) and superfused; subsequent to a 60 min washout period, 4 min fractions of superfusate were collected. Different types of stimulation were applied in minutes 76 and 96. [^3^H] overflow was either triggered by electrical field stimulations (EFS; 60 monophasic rectangular pulses at 1 Hz), 40 mM K^+^, or 0.5 mM sucrose. Panel **(A)** shows the time-course of [^3^H] outflow in typical experiments using these various stimulation paradigms, as indicated. From minute 84 onward, either solvent (empty circles), H_2_S (1 mM) or NaHS (1 mM, filled circles) were present (*n* = 3). **(B)** Effects of H_2_S (1 mM) and different concentrations of NaHS (0.01 to 1 mM, as indicated) on noradrenaline release evoked by either electrical field stimulation (*n* = 12), K^+^ (*n* = 12), or sucrose (*n* = 9). ^*^, ^**^, and ^***^ Indicate significant differences vs. solvent at *p* < 0.05, *p* < 0.01, and *p* < 0.001, respectively (Kruskal-Wallis test, Dunn's Multiple comparison test).

After just 1 week in culture, SCG neurons release noradrenaline rather than acetylcholine (Landis, 1990). Exocytotic release of noradrenaline in such cultures occurs exclusively at the nerve terminals (Boehm, 1999) which are located within the target organs, such as arteries, under native conditions. To elucidate whether H_2_S might exert direct effects on the output of sympathetic neurons, 1-week old cultures were loaded with [^3^H]-noradrenaline ([^3^H]-NA) as radiotracer. Release of radioactivity as readout for noradrenaline was triggered by electrical field stimulations, 40 mM K^+^, or 0.5 M sucrose. These stimulation paradigms elicit action potential propagation, membrane depolarization independently of action potentials, and exocytosis due to hyperosmolarity, respectively. NaHS at 0.01 to 1 mM increased stimulation-evoked tritium release in a concentration-dependent manner irrespective of the stimulus being employed (Figures 7A,B). H_2_S mimicked the effect of the H_2_S donor on electrically evoked release. Thus, H_2_S may enhance the output of sympathetic neurons within target tissues.

## 4. Discussion

Despite the extensive knowledge on H_2_S as a regulator of blood pressure (Meng et al., 2014; van Goor et al., 2016) and the well-known role of the sympathetic nervous system in blood pressure control (Guyenet, 2006), effects of H_2_S on neurons within sympathetic ganglia remained largely unexplored. Even though evidence for endogenous synthesis of H_2_S in sympathetic ganglia has been presented (Sha et al., 2013), it is still unknown how this gasotransmitter may impinge on the excitability of ganglionic neurons. The present results reveal that H_2_S as well as the H_2_S donor NaHS, both in the submillimolar range, cause hyperpolarization of SCG neurons and reduce action potential firing. The concentrations of NaHS that were required to achieve effects equivalent to those of H_2_S were approximately threefold higher. This agrees well with the fact that the H_2_S concentrations liberated from NaHS amount to about 25% of that of the donor itself (Sitdikova et al., 2014). We therefore infer that the actions of NaHS as detected in the present experiments were all mediated by H_2_S.

The H_2_S -dependent hyperpolarization in SCG neurons is in sharp contrast to what has been learned about actions of this gasotransmitter in the peripheral nervous system so far: in sensory neurons of trigeminal (Feng et al., 2013) and dorsal root (Lu et al., 2014) ganglia, NaHS has been reported to elicit depolarization and to increase membrane excitability. Those effects were suggested to involve suppression of a sustained depolarization-evoked K^+^ current (Feng et al., 2013), on the one hand, and activation of TRPV1 channels (Lu et al., 2014) on the other hand. As sympathetic neurons in general and SCG neurons in particular do not express TRPV1 channels (Wood et al., 1988), the latter mechanism is irrelevant for the present experiments. However, sustained depolarization-evoked K^+^ currents in trigeminal neurons are partially reduced in the presence of linopirdine, a selective blocker of K_υ_7 channels (Abd-Elsayed et al., 2015). Hence, the K^+^ currents reduced by NaHS in trigeminal neurons are likely to involve K_υ_7 channels. Likewise, in the present experiments 1 mM NaHS reduced non-inactivating outward currents in SCG neurons determined at a potential of −30 mV as well as deactivation currents observed upon hyperpolarization of the membrane to −55 mV which are mainly carried by K_υ_7 channels (Boehm, 1998). Thus, high NaHS concentrations did reduce currents through K_υ_7 channels, and this mechanism must be expected to slightly depolarize the neurons and to increase action potential firing (Jones et al., 1995). Interestingly, millimolar concentrations of NaHS have been reported to enhance rather than reduce currents through recombinant human K_υ_7.2/7.3 channels (Di Cesare Mannelli et al., 2017). Whether this discrepancy vs. the present results obtained with native rat K_υ_7 channels expressed in SCG neurons [which are most likely composed of K_υ_7.2/7.3 heteromers; (Wang et al., 1998)] can be explained by either species differences or variations between native and recombinant systems remains open for future investigations.

Membrane potential and excitability of sympathetic neurons varies between different ganglia and is mainly controlled by the magnitude of various K^+^ currents which also determine size and occurrence of afterhyperpolarizations and afterdepolarizations, respectively (Jobling and Gibbins, 1999). Up to now, some of the K^+^ channels that underlie these currents have been identified. In particular, K_υ_7 and K_*Ca*_1 channels have been documented to represent major determinants of membrane excitability in SCG neurons (Jones et al., 1995; Vivas et al., 2014). Recently, Ca^2+^ activated Cl^−^ channels have been evidenced to contribute to the regulation of excitability in SCG neurons as well (Salzer et al., 2014). At membrane voltages corresponding to the resting membrane potential of SCG neurons, NaHS triggered outward currents that were (i) similar to currents elicited by diazoxide and (ii) blocked by tolbutamide, activators and inhibitors of K_*ATP*_ channels, respectively (Foster and Coetzee, 2015). Since H_2_S is well-known as an opener of the latter type of channel (Wang, 2012), this indicates, for the first time, that K_*ATP*_ channels may contribute to the control of membrane potential and excitability in sympathetic ganglion cells. The lack of effect of tolbutamide alone on the membrane potential indicates that K_*ATP*_ channels are closed under resting conditions. This may be the reason why their contribution to membrane potential regulation has not been appreciated before. Nevertheless, opening of K_*ATP*_ channels, whether by NaHS or diazoxide was found to be sufficient to cause hyperpolarization and to reduce action potential firing in SCG neurons. Taken together, these results reveal the opening of K_*ATP*_ channels as a mechanism to reduce the excitability of neurons in sympathetic ganglia.

In mouse mesenteric ganglia, H_2_S was shown to enhance cholinergic transmission in an input-specific manner, i.e., depending on which afferent fibres were stimulated in order to evoke postsynaptic responses (Sha et al., 2013). In long-term cultures, SCG neurons form cholinergic synapses (O'Lague et al., 1974), and frequencies, but not amplitudes, of mEPSCs at such synapses were enhanced by NaHS. This points toward a presynaptic site of action of NaHS, and this was corroborated by a lack of effect of the H_2_S donor on currents through nAChRs. Similar results have been obtained at mouse neuromuscular junctions where frequencies, but not amplitudes of miniature endplates potentials were increased in the presence of NaHS (Gerasimova et al., 2015).

An akin presynaptic facilitation of transmitter release by NaHS was also detected by measurements of noradrenaline release from short-term SCG cultures. This enhancing action on noradrenaline release did occur irrespective of the stimulation paradigm employed to trigger exocytosis, and was even seen when release was elicited by high sucrose in a Ca^2+^-independent manner (Rosenmund and Stevens, 1996). Thus, NaHS acted downstream of Ca^2+^ entry on some step involved in vesicle exocytosis in order to enhance sympathetic transmitter release. In contrast to the present results, noradrenaline release from tissues innervated by postganglionic sympathetic axons, such as iris-ciliary bodies, has been found to be reduced by H_2_S donors (Kulkarni et al., 2009; Salvi et al., 2015). This latter effect was shown to be counteracted by blockers of K_*ATP*_ channels (Salvi et al., 2015). A major difference between such tissue preparations and dissociated cultures of sympathetic ganglia is the presence vs. the lack of target organs. It is well-known that (i) any mediator derived from the target tissue may influence noradrenaline release and (ii) within target organs sympathetic transmitter release is subject to a huge autoinhibitory feedback tone (Boehm and Kubista, 2002). Moreover, in iris-ciliary bodies application of H_2_S donors will add to the level of endogenous H_2_S within the tissue samples and the final concentrations that are achieved remain unknown (Kulkarni et al., 2009). In the present experiments, in contrast, endogenous H_2_S can hardly play any role as the monolayer cultures are continuously superfused by physiological buffer. Taken together, results obtained in primary cultures as shown above reveal the direct and singular action of H_2_S on sympathetic neurons, whereas those from sympathetically innervated tissues reflect the more complex situation *in situ*.

In the present experiments, H_2_S and/or H_2_S donors were found to enhance release of both, acetylcholine and noradrenaline. Previously, NaHS has been shown to enhance glutamate release from central neurons (Austgen et al., 2011) and acetylcholine release from motor neurons (Gerasimova et al., 2015). Potential underlying mechanisms have been investigated in experiments determining the outflow of [^3^H]-NA previously taken up by the neurons. NaHS did not only enhance noradrenaline release triggered by either electrical field stimulation or K^+^ depolarization which both rely on transmembrane Ca^2+^ entry via voltage-gated Ca_*v*_2 channels (Boehm and Kubista, 2002), but also that evoked by 0.5 mM sucrose which causes Ca^2+^-independent exocytosis of vesicles within the readily releasable pool that are also recruited by action potentials (Rosenmund and Stevens, 1996). Hence, H_2_S must have acted downstream of action potentials and Ca^2+^ entry via Ca_*v*_2 channels, and this also excludes K_*ATP*_ channels from the underlying mechanisms. At the neuromuscular junction, H_2_S had been suggested to cause presynaptic facilitation through cyclic AMP-dependent mechanisms (Gerasimova et al., 2015) which also determine noradrenaline release from sympathetic nerve terminals (Boehm and Kubista, 2002). Nevertheless, the precise site of action of H_2_S with respect to the enhancement of transmitter release from sympathetic neurons remains to be determined.

In summary, the present results reveal that H_2_S exerts direct actions on sympathetic neurons that vary between diverse subcellular compartments: at the somatodendritic region, the membrane potential is hyperpolarized and action potential firing is impeded by the gasotransmitter, both due to the opening of K_*ATP*_ channels; in contrast, noradrenaline release from nerve terminals is enhanced by H_2_S through a Ca^2+^-independent action on exocytosis. In addition, the input from preganglionic neurons is increased through a presynaptic action of H_2_S on acetylcholine release (Sha et al., 2013). Therefore, the inhibitory action of H_2_S donors on the vasopressor response elicited by preganglionic stimulation of the sympathetic nervous system (Centurión et al., 2016) most likely involves a decrease in membrane excitability caused by an activation of K_*ATP*_ channels.

## Author contributions

MD, HD acquired and analyzed data, and revised the manuscript. SB conceived and designed the project, interpreted the data and drafted and revised the manuscript. IS designed experiments, interpreted the data and drafted and revised the manuscript. All authors have read and approved the manuscript.

### Conflict of interest statement

Conflict of Interest Statement:The authors declare that the research was conducted in the absence of any commercial or financial relationships that could be construed as a potential conflict of interest.
